# Editorial: NK Cell-Based Cancer Immunotherapy

**DOI:** 10.3389/fimmu.2016.00249

**Published:** 2016-06-27

**Authors:** Francisco Borrego, Susana Larrucea, Rafael Solana, Raquel Tarazona

**Affiliations:** ^1^BioCruces Health Research Institute, Cruces University Hospital, Barakaldo, Spain; ^2^Ikerbasque, Basque Foundation for Science, Bilbao, Spain; ^3^Instituto Maimónides de Investigación Biomédica de Córdoba (IMIBIC), Reina Sofía University Hospital, University of Córdoba, Córdoba, Spain; ^4^University of Extremadura, Cáceres, Spain

**Keywords:** NK cells, adoptive cell therapy, cancer immunotherapy, ADCC, cytokines, CAR, NK-92

**The Editorial on the Research Topic**

**NK cell-based cancer immunotherapy**

Innate and adaptive immunity cooperate to eliminate tumors. However, when infrequent cancer cell variants are not destroyed, tumor growth and immunosurveillance enter into a dynamic equilibrium until cancer cells evade the immune system, at which point malignancies appear clinically as a consequence. Therapies designed to induce potent antitumor responses by harnessing the power of the immune system are an appealing strategy to control tumor growth. Natural killer (NK) cells are innate lymphocytes that play a pivotal role in host immunity against cancer. The activity of NK cells is finely tuned by the balance between the signals that emanate from inhibitory and activating receptors. Inhibitory receptors, such as killer-cell immunoglobulin-like receptor (KIR) and CD94/NK Group 2 member A (NKG2A), recognize human leukocyte (HLA) class I molecules whose expression is often altered on tumor cells. NK cells recognize tumor cells by activating receptors, such as natural cytotoxicity receptors (NCRs) and NKG2D, which sense the changed expression of their ligands on the cancer cell surface. Providing important insights, the past 15 years have witnessed an explosion of research into the biology and clinical applications of NK cells. Current NK cell-based cancer immunotherapy aims to reverse the tumor-induced NK cell dysfunction that is observed in patients with cancer and to increase and sustain NK cell effector functions. Therapies involving NK cells may either activate endogenous NK cells or involve transfer of exogenous cells by hematopoietic stem cell transplantation (HSCT) or adoptive cell therapy.

In this research topic, we have collected several articles that highlight the exciting potential that NK cells exhibit as an effective tool in cancer immunotherapy. We open the research topic with two articles that describe NK cell surface receptors involved in the recognition of tumor target cells. Chester et al. briefly describe NK-cell–tumor interactions and the three most important mechanisms of how NK cells kill target cells, i.e., natural killing, antibody-dependent cell-mediated cytotoxicity (ADCC), and death receptor-induced apoptosis, and follow with a description of the best studied activating and inhibitory receptors involved in tumor cell recognition, along with the ability of agonistic monoclonal antibodies (mAbs) specific for costimulatory molecules, such as CD137 and OX40. Horton and Mathew focus their review on NCRs, with a special attention to NKp44 and its dual activating and inhibitory function following recognition of different ligands. They suggest that NCRs serve as receptors for damage-associated molecular pattern (DAMP) molecules in association with HLA class I molecules, heparan sulfate proteoglycans (HSPGs), or other coligands and, therefore, regulate NK cell activity. A better understanding of the tumor immunosuppressive microenvironment is very important to design efficient NK cell-based therapies. Hasmim et al. review the cell types within the tumor that are involved in the suppression of NK cells, including M2-polarized macrophages, myeloid-derived suppressor cells (MDSC), regulatory T cells (Treg), and fibroblasts, and how the microenvironmental hypoxia that is characteristic of solid tumors inhibit NK cell functions.

Next, there is a group of articles that provide a general vision of how to obtain and harness NK cells to fight tumors. Dahlberg et al., Domogala et al., Pittari et al., and Rezvani and Rouce have reviewed the different cellular sources and methods to isolate, differentiate, genetically engineer, expand, and activate *ex vivo* and *in vivo* NK cells, including autologous and allogeneic NK cells and NK cell lines. In addition to NK cell lines, sources to generate NK cell products include NK cells from peripheral blood or from cord blood and NK cells differentiated from CD34^+^ hematopoietic precursors or pluripotent stem cells (embryonic stem cells or induced pluripotent stem cells). In general, adoptive NK cell-based therapy has been more successful in the treatment of hematological tumors than in patients with solid tumors, and the use of tools aimed to reverse the immunosuppressive tumor microenvironment significantly will improve the efficacy of this type of therapy. A cell product termed cytokine-induced killer (CIK) cells, which possess phenotypic and functional features of both NK cells and T cells, is also described by Pittari et al.

Similar to T cells, genetic manipulation of NK cells is emerging as a promising tool to increase their antitumor activity. Chimeric antigen receptors (CARs) consist of an extracellular domain, generally a small chain variable fragment, specific for a tumor antigen that is linked with one or more intracellular domains able to induce activation signals. In this study, Hermanson and Kaufman extensively review the CAR constructs with different intracellular activation domains that have been described, to date, in NK cells from several sources, suggesting that depending on the tumor type and/or target antigen, different CAR constructs may be required for optimal activation of NK cells. Carlsten and Childs review the advantages and challenges of methods to genetically modify NK cells and give an overview of different strategies to reprogram NK cells with the objective to improve the persistence and expansion of infused cells, to enhance the migration to the tumors, and to improve their cytotoxicity. Cell lines, such as NK-92, could be an alternative to NK cells from patients or allogeneic donors. They could easily be expanded in culture, genetically manipulated, and may represent an off-the-shelf product ready for use. Klingemann et al. review that NK-92 is the only cell line that has been studied in clinical trials with clinically significant responses and minimal adverse reactions.

In contrast to normal cells, many tumor cells express heat-shock protein 70 (Hsp70) at the cell surface and get released into the circulation. Membrane Hsp70 (mHsp70) correlates with high aggressiveness of the tumors. In this research topic, Gunther et al. describe that patients with squamous cell and adeno non-small cell lung cancer (NSCLC) exhibited high levels of serum Hsp70. Furthermore, they found a positive correlation between serum levels of Hsp70 with gross tumor volume and an inverse correlation with CD69^+^/CD94^+^ NK cells in squamous NSCLC, suggesting that activated NK cells somehow may be involved in the control of tumor growth. The same group has previously found in preclinical studies that NK cells activated with a naturally occurring Hsp70 peptide (TKD) and IL-2 are able of specifically kill mHsp70-expressing tumors, but not mHsp70 negative ones. Here, they summarize a Phase I clinical trial of TKD/IL-2-stimulated autologous NK cells with NSCLC and describe an ongoing Phase II clinical trial of TKD/IL-2-stimulated NK cells for the treatment of patients with NSCLC, following radiochemotherapy (Specht et al.).

The mechanism of action of many therapeutic mAbs for cancer treatment involves, at least partially, ADCC through FcγRIIIA/CD16a. Many studies have shown that the clinical outcome after treatment of patients with mAbs is correlated with polymorphisms at the *FCGR3A* gene, which encodes for FcγRIIIA/CD16a, that affect the binding affinity to mAbs. Wang et al. review some of the current therapeutic mAbs that are being used in the clinic and strategies that increase their ADCC, such as modifying the glycosylation patterns of the mAbs, combining them with other mAbs, radiation therapy, matrix metalloproteases inhibitors or cytokines, and by designing new molecular entities such as immunocytokines and bi-specific antibodies. Cetuximab, an anti-epidermal growth factor receptor (EGFR) mAb, exerts ADCC against EGFR^+^ target cells. Kloss et al. show that patients with head and neck squamous cell carcinomas (HNSCCs) have elevated levels of soluble major histocompatibility complex class I chain-related peptide A (sMICA) and transforming growth factor beta 1 (TGF-β1) in serum, which are responsible for the impaired NK cell effector functions and decreased NKG2D expression. They show that cetuximab restores the NK cell-mediated killing of sMICA-inhibited patient NK cells against HNSCC cells *via* ADCC and enhances tumor infiltration of NK cells in HNSCC tumor spheroids.

Autologous hematopoietic stem cell transplantation (autoHSCT) is a therapeutic indication for multiple myeloma or malignant lymphoma, and it has been shown that the reconstitution levels of the NK cell pool after autoHSCT has a prognostic value. Jacobs et al. have studied the phenotype and function of NK cells after autoHSCT. They found that CD56^++^ NK cells were the major subset 1–2 days after leukocyte regeneration (>1000 leukocytes/μl) and that is characterized by a high expression of CD57 and KIRs, which is age dependent, and that are able to degranulate and produce cytokines after tumor interaction. On the other hand, preclinical and clinical data have demonstrated that, in the context of haploidentical T-cell-depleted HSCT, alloreactive NK cells are able to exert a very important antitumor activity, with no increased incidence of GVDH, and that mature alloreactive NK cells can be safely infused into patients. The KIR–HLA class I mismatch between donor and recipient in the graft versus leukemia (GVL) direction has demonstrated to enhance the antitumor activity of NK cells. In this research topic, two articles by Lim et al. and Ruggeri et al. review the present and future of alloreactive NK cells for tumor treatment, mostly acute myeloid leukemia (AML), in the context of allogeneic HSCT and infusions of alloreactive purified NK cells. These are emerging as safe and potent effectors against tumors.

Based on their expressed pattern of cell surface receptors, NK cells are divided in subsets that are able to mediate different effector functions and are characterized by distinct homing properties. Gismondi et al. have reviewed this issue, suggesting that, for improved and more efficient NK cell-based therapies, it is necessary to identify, isolate, expand, and administer NK cell subsets that exhibit increased effector functions and have the adequate homing capabilities to reach the tumors. Chretien et al., with an automated procedure using the FLOCK algorithm and a panel of three markers (CD56, CD57, and KIRs), define five maturation stages of NK cells from human peripheral blood. By analyzing a cohort of healthy volunteers and another cohort of patients with AML, they found that the latter displayed marked differences compared with healthy donors. Moreover, within the AML cohort, it was possible to define three distinct groups of patients according to their maturation profiles, which might be useful for prognostic purposes. Tarazona et al. have reviewed the current knowledge about the role of NK cells on the recognition and elimination of melanoma cells and the strategies against melanoma based on NK cells. *In vitro* experiments, *in vivo* data from murine models, and observations from melanoma patients indicate that NK cells have a role in the immune response against melanoma. NK cell-based therapies against melanoma include, among others, modulation of NK cell responses by administration of cytokines, treatment with checkpoint inhibitors and bi-specific antibodies, and by adoptive NK cell therapy with autologous or allogeneic NK cells and NK cell lines, genetically modified or not.

Finally, we want to express our gratitude to all the authors who have contributed to this research topic and to the reviewers for their magnificent job. We hope that the reader will find this research topic motivating and helpful. We invite you to read the following articles and immerse yourself in the interesting world of NK cell-based cancer immunotherapy.

## Author Contributions

All authors listed, have made substantial, direct and intellectual contribution to the work, and approved it for publication.

## Conflict of Interest Statement

The authors declare that the research was conducted in the absence of any commercial or financial relationships that could be construed as a potential conflict of interest.

